# Tomato Anomalies Detection in Greenhouse Scenarios Based on YOLO-Dense

**DOI:** 10.3389/fpls.2021.634103

**Published:** 2021-04-09

**Authors:** Xuewei Wang, Jun Liu

**Affiliations:** Shandong Provincial University Laboratory for Protected Horticulture, Blockchain Laboratory of Agricultural Vegetables, Weifang University of Science and Technology, Weifang, China

**Keywords:** deep learning, plant diseases recognition, DenseNet, real-field scenarios, object detection

## Abstract

Greenhouse cultivation can improve crop yield and quality, and it not only solves people’s daily needs but also brings considerable gains to the agricultural staff. One of the most widely cultivated greenhouse crops is tomato, mainly because of its high nutritional value and its good taste. However, there are a number of anomalies for the tomato crop that pose a threat for its greenhouse cultivation. Detection of tomato anomalies in the complex natural environment is an important research direction in the field of plant science. Automated identification of tomato anomalies is still a challenging task because of its small size and complex background. To solve the problem of tomato anomaly detection in the complex natural environment, a novel YOLO-Dense was proposed based on a one-stage deep detection YOLO framework. By adding a dense connection module in the network architecture, the network inference speed of the proposed model can be effectively improved. By using the K-means algorithm to cluster the anchor box, nine different sizes of anchor boxes with potential objects to be identified were obtained. The multiscale training strategy was adopted to improve the recognition accuracy of objects at different scales. The experimental results show that the mAP and detection time of a single image of the YOLO-Dense network is 96.41% and 20.28 ms, respectively. Compared with SSD, Faster R-CNN, and the original YOLOv3 network, the YOLO-Dense model achieved the best performance in tomato anomaly detection under a complex natural environment.

## Introduction

With the development of the economy, agriculture is transforming from traditional to modern, and greenhouses, as an important support for modern agriculture, are widely used. A greenhouse can be kept untouched by the external environment and at the same time not be restricted by the geographical and seasonal factors of each crop cultivation, thus showing the capabilities of controlled environment agricultural production (Xie et al., 2017). Tomato, an important vegetable variety grown in greenhouses, is one of the most prevalent fruits and vegetables cultivated worldwide. Tomato is highly favored by global consumers because it is rich in antioxidant lycopene, multivitamins, and minerals and has the advantages of low heat and considerable cultivation benefits (Heuvelink, 2005). However, a research field with huge potential about tomato cultivation is dealing with the detection of possible threats because crop diseases are one of the main factors affecting the yield and quality of agricultural products.

Diseased crops usually show discoloration, necrosis, deformity, decay, and wilting after infection (Alemu, 2015). Most of the disease phenomena will be reflected in the leaves of crops (Pethybridge and Nelson, 2015), and the judgment of diseases through the leaves of crops has become one of the common means in the field of agriculture (Barbedo, 2016). Traditional identification of crop diseases is accomplished manually by farmers in the field, and incorrect diagnosis and unnecessary pesticide application are common (Juncheng et al., 2018). Not only that, the traditional manual judgment method has the disadvantages of being time-consuming and labor-intensive, and human-subjective factors play a major role (Ghazi et al., 2017).

China is one of the largest tomato-producing and -consuming countries in the world (Xu et al., 2000), and tomato production is one of the important ways for farmers to increase their income (Liu, 2018). Early detection of tomato anomalies in the complex natural environment is of great significance to reduce the cost of manual identification and improve tomato quality and yield. The primary task and design difficulty are the real-time and accurate identification and spatial detection of tomato anomalies. The growth stages of tomato plants present high diversification, and tomato anomaly images in natural environments can be easily influenced by light, occlusion, and background noise, which cause some difficulties in the detection of tomato anomalies. Therefore, the rapid and accurate identification of tomato anomalies in the complex environment is a key problem in achieving an automated inspection of tomato anomalies.

When using object detection algorithms, usually feature extractors such as histogram of gradient (HOG), scale-invariant feature transform (SIFT), and Haar-like feature are manually designed to extract object features, which are then given as input to the support vector machine (SVM), iterator AdaBoost, random forest (RF), and other classifiers for classification and recognition. The most common object detection methods for tomato anomalies are basically based on color and shape features for feature extraction and analysis (Xu et al., 2011; Martinelli et al., 2015). The generalization of the most common method is poor, and it is difficult to extract reasonable features. Also, the calculation complexity is high, and therefore sometimes the requirements of accuracy and speed for real-time detection are not fulfilled. Most of them do not take into account the influencing factors in the complex environment of a greenhouse and have insufficient robustness against the changes of various features, so it is difficult to meet the actual requirements. Deep convolutional neural networks (DCNNs), which have emerged in recent years, provide a new idea for tomato anomaly object detection.

Deep learning-based detection algorithms can be divided into region-based and regression-based. The region-based method generates candidate regions by selective search algorithm and then classifies them using convolutional neural networks. A few of these methods are RCNN (Girshick et al., 2014), Fast R-CNN (Shahid et al., 2015), Faster R-CNN (Ren et al., 2017), and SPP-Net (He et al., 2014). This kind of region-based, two-step method can achieve high detection accuracy, but it has the disadvantages of complex network and slow detection speed. Regression-based methods such as SSD (Liu et al., 2016) and YOLO (Redmon et al., 2016) frame take the object detection problem as a regression one, so the object class probability and position coordinates can be directly regressed. The YOLO series algorithm based on regression have fast processing speed and high accuracy, so they have been widely used in actual scenarios, such as fruit detection (Xu et al., 2020), mask-wearing detection (Ren and Liu, 2020), and traffic sign detection (Zhang et al., 2021). YOLOv2 (Redmon and Farhadi, 2017) and YOLOv3 (Li Y. et al., 2019) were improved on the basis of the YOLO algorithm, which further enhances the detection effect. However, the network structure of the fast regression-based detection algorithm remains large, and therefore the speed of deploying to embedded services is slow while the deployment cost is high.

Artificial intelligence has been widely used in agriculture in recent years (Tang et al., 2020). Farmers have gradually begun to use smartphones to detect crop anomalies (Prasad et al., 2014). In view of the problem of crop anomaly detection, Li W. et al. (2019) proposed a pipeline based on deep learning for locating and counting agricultural pests in image by a self-learning saliency feature map, and the average accuracy (mAP) achieved was 0.885. Xing et al. (2019) developed a simple but effective CNN model based on a self-built dataset, which increased the complexity of cross-channel operation and made the frequency of feature reuse adapt to the network depth, but this algorithm cannot be widely used in systems of general performance as it requires a high amount of computation. For the detection of tomato anomalies, Fuentes et al. (2018) proposed a detection algorithm in a real, natural environment based on Faster R-CNN, and the average accuracy it achieved was up to 96% for 10 common tomato anomalies including leaf mold, gray mold, canker, plague, miner, low temperature, powdery mildew, whitefly, nutritional excess, and yellow leaf curl. Zhang Y. et al. (2020) also proposed a detection method based on the improved Faster R-CNN algorithm, but this method has a slow detection speed and is not suitable for the real-time detection of tomato anomalies with large datasets.

YOLOv3 is an end-to-end object detection algorithm based on Darknet-53, and multiscale feature fusion is done via FPN (Feature Pyramid Networks) (Lin et al., 2017). YOLOv3 has the characteristics of fast detection speed and strong comprehensive performance, but when it is directly applied to certain specific detection objects, due to the influence of scene complexity and feature diversification, the detection effect cannot meet the requirements, so it needs to be improved. Zhang X. et al. (2020) improved YOLOv3 using an expanded spatial pyramid, achieving a speed of 56 frames/second and an average accuracy of 82.2%. Chen et al. (2020) designed an anchor box (a set of initial prediction boxes with fixed width and height, and the number and width of the anchor boxes clustered by different datasets are different) that was more suitable for face detection, and a new loss function was used to replace the square sum loss function, which reduced the model error and improved the convergence speed. Fu et al. (2020) automatically detected kiwifruit in orchards by improving the YOLOv3-tiny model; two convolution cores of 3 × 3 and 1 × 1 were added to the fifth and sixth convolution layers of the YOLOv3-tiny model to develop the deep YOLOv3-tiny (DY3TNet) model. The experimental results showed that the improved DY3TNet model had small volume and reduced computational complexity, thus realizing real-time detection. Xu et al. (2020) improved YOLOv3 by using soft-NMS (non-maximum suppression) instead of NMS to reduce the loss of the prediction bounding box due to green mango overlap, which can meet the requirements of real-time detection for robotic picking.

The objective of this study is to introduce the idea of dense connection in DenseNet (Dense Convolutional Network) (Huang et al., 2016) into a YOLOv3 basic network, and a YOLO-Dense object detection algorithm is proposed. DenseNet is a densely connected network structure. All layers in the network are directly connected. The input of each layer in the network is the intersection of all the output layers in front, and the feature map learned in each layer of the network will also be directly transmitted to the latter layer as the output, so the multiplexing of features can be enhanced. The improved method begins by designing the YOLO Dense network based on the structure of Darknet-53 in YOLOv3. Then, the improved K -means clustering algorithm was used for calculating the anchor bounding boxes, in order to reduce the impact of the initial points on the clustering results. Finally, the last step of the method involves the multiscale training of the network.

## Method for Improving the Yolov3 Model

**FIGURE 1:**
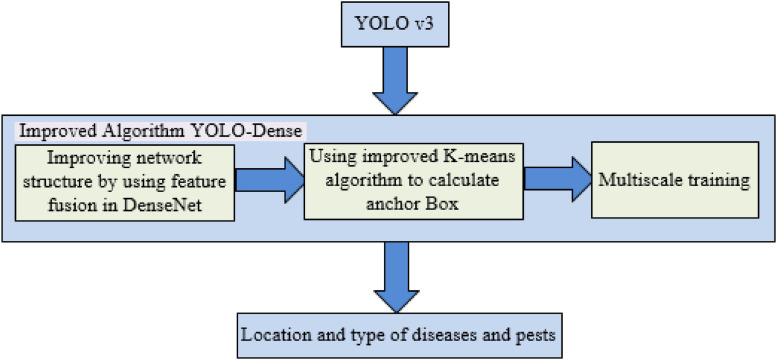
Schematic diagram for improving the YOLOv3 model 2.1 YOLO-Dense network design.

YOLOv3 has excellent detection effects in the field of object detection, but for the detection task of tomato anomalies in the complex natural environment, the network needs to be improved. In this study, an improved YOLO-Dense network model was proposed based on the characteristics of the self-made tomato anomaly dataset. The improvement scheme is shown in Figure 1.

In the YOLOv3 network structure, the Darknet-53 network was used to extract features. Due to the increase in the number of layers in the network, it is possible that an overfitting or gradient (diffusion, explosion) problem will occur. Therefore, the idea of residual network is used in the Darknet-53 network: the original output of a layer is directly connected to the back layer, and the residual layer is constructed between layers of the same dimension. The problem of gradient disappearance in deep neural network is solved by means of Jump-Layer connection.

This study draws on a DenseNet network idea: the input of each layer in the network is the sum of the output of all the layers in front, and the output of this layer will also propagate backward, becoming part of the input of the latter layer. The new YOLO-Dense network, which uses dense connection, can realize feature fusion through dimension connection on the channel, which is helpful for feature extraction of tomato anomalies. Meanwhile, it reduces parameters and calculation costs and improves network efficiency. Therefore, it is necessary to change part of the residual layer in the underlying network Darknet-53 of YOLOv3 into a densely connected network and refer to the DenseNet network for naming. The modified structure can make more effective use of the features extracted in the prediction layer, and thus its detection speed is faster than the original YOLOv3.

**FIGURE 2:**
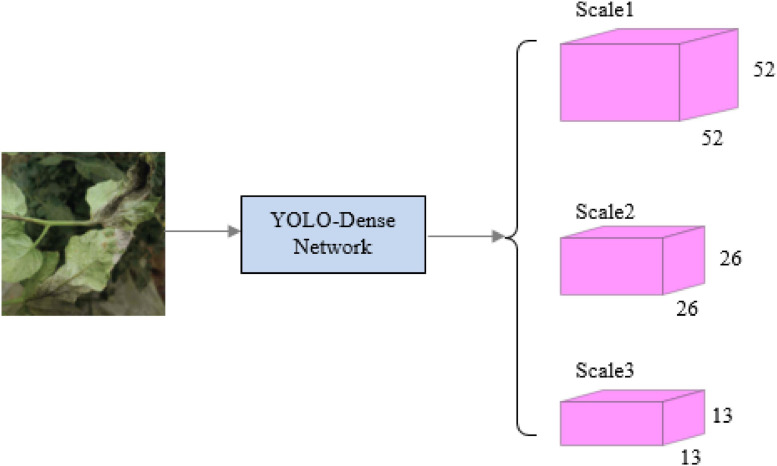
The system structure.

Figure 2 shows a descriptive graphical depiction of the YOLO-dense on how the system works.

**FIGURE 3:**
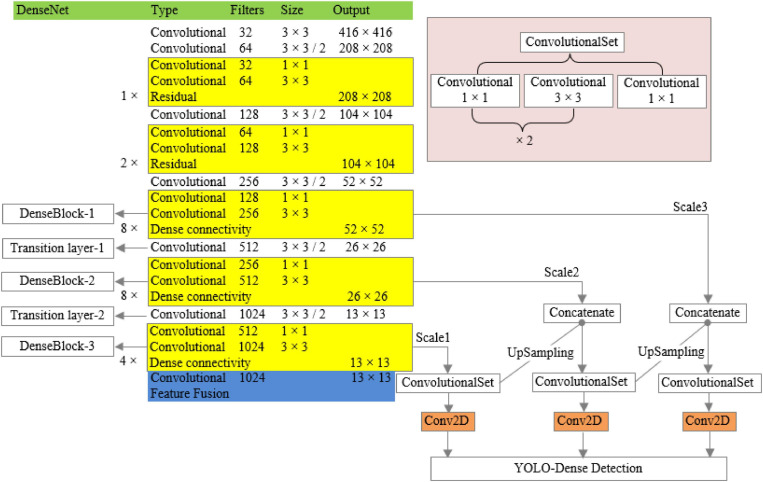
YOLO-Dense network model.

The improved network structure model is shown in Figure 3 below:

The specific ways of improvement are as follows: the dimensions of the 13th layer to the 36th layer of Darknet-53 are the same, and there is no need to be transformed, so the residual layer in Darknet-53 is changed to the dense connection layer, and the shortcut layers in the 15th, 18th, 21st, 24th, 27th, 30th, 33rd, and 36th layers of Darknet-53 are changed to route layers. The original residual layer is changed to dense connection layer to achieve the dense connection of the features of the same dimension, and the design was named DenseBlock-1. The shortcut layers of Layers 40, 43, 46, 49, 52, 55, 58, and 61 were changed to route layers, the original residual layer was changed to dense connection layer, and the design was named DenseBlock-2. Similarly, the shortcut layers of Layers 65, 68, 71, and 74 were changed to route layers, and the original residual layer was changed to dense connection layer, and the design is named DenseBlock-3. The 37-layer convolution layer in Darknet-53 is similar to the transition layer of the DenseNet network in function, both of which reduce the dimensionality of the output feature map, so the 37-layer layer is renamed Transitionlayer-1; similarly, the 62-layer layer is renamed Transitionlayer-2.

**FIGURE 4:**
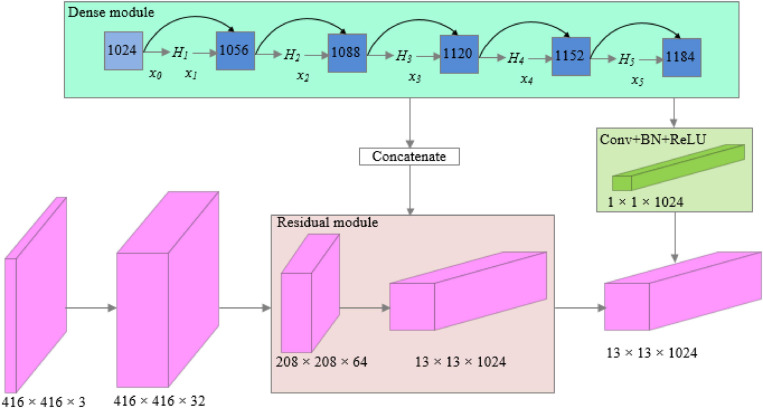
Structure of dense modules of the YOLO-Dense network.

The structure of dense modules of the improved network is shown in Figure 4, which can realize multilayer feature multiplexing and fusion and avoid the computational complexity caused by the new structure.

In the dense module:

(1) $$x_{l}\,H_{l}\,\left(\left[x_{0},x_{1},\cdots ,x_{l-1}\right]\right),l\,1,2,3,4,5$$

In the abovementioned formula, and *x_0* is the input feature map of the module, *x_1* is the output of the first layer. [*x*_0_,*x*_1_,⋯,*x*_*l*−1_] is the concatenation of *x*_0_,*x*_1_,⋯,*x*_*l*−1_. *H*_*l*_([*x*_0_,*x*_1_,⋯,*x*_*l*−1_]) is the combination function of BN (batch normalization), ReLU (rectified linear units), and convolution, to realize the *l* layer nonlinear transformation.

The YOLO-Dense network uses the YOLO detection layer for class output and uses the three different prediction scales to detect objects of different sizes, with different prediction scales of 13 × 13, 26 × 26, and 52 × 52, respectively. The predictive scale output feature map has two sets of dimensions: the dimension of extracted features, such as 13 × 13, and the second dimension is calculated by using the following formula:

(2) d⁢i⁢m⁢e⁢n⁢s⁢i⁢o⁢n⁢a⁢l⁢i⁢t⁢y+B×(5+C)

In the abovementioned formula, B indicates the number of bounding boxes per prediction and C is the number of classes of the bounding boxes. So, another dimension is 3 × (5+6) = 33. In the output layer of the network, the Softmax classifier used in the original YOLO network cannot identify and locate two kinds of anomalies in the same grid correctly. Therefore, this study uses a separate logistic classifier for each different disease category to predict the confidence score that each anchor box belongs to a specific category and replaces the original Softmax classifier with it.

### Anchor Box Calculation Using the Improved K-Means Clustering Algorithm

YOLOv3 borrows the idea of using the anchor box in Faster R-CNN. The anchor box is used as *a priori* box to assist in predicting the object bounding box, which is designed according to different datasets. For the self-built dataset in this study, the anchor box needs to be recalculated.

The K-means algorithm usually uses Euclidean distance, Chebyshev distance, Manhattan distance, and other methods as distance measures to calculate the distance between two points. YOLO v3 has used the K-means clustering algorithm and got nine prediction boxes. The K-means clustering algorithm of YOLO v3 is based on features from the PASCAL-VOC dataset, which in turn produces a prediction box, and since images in the PASCAL-VOC dataset have large gaps from tomato anomaly features, it has poor detection accuracy for tomato anomalies and is not suitable for tomato anomaly detection tasks. In addition, the K-means clustering algorithm randomly chooses K points as the initial clustering center (i.e., there are k classifications), and this random way increases the randomness of the clustering and affects the clustering effect of the algorithm. In this study, if the clustering algorithm uses these commonly used distances, the larger the candidate box generated, the greater the error, so it will not produce good detection results. The main focus of this study is small object detection; thus, the original anchor is not applicable. It is necessary to find a more suitable anchor box by the clustering algorithm, which can help improve the average accuracy and speed of small-object detection. Considering that the K-means algorithm is sensitive to the initial value setting, and it is easy to converge to the local optimum when the dataset is large, this study uses the K-means + + (Arthur and Vassilvitskii, 2007) algorithm to obtain the initial value before clustering. Therefore, the similarity between bounding boxes can be calculated by a custom distance formula, which is as follows:

(3) d⁢(b⁢o⁢x,c⁢e⁢n⁢t⁢r⁢o⁢i⁢d)=1-I⁢o⁢U⁢(b⁢o⁢x,c⁢e⁢n⁢t⁢r⁢o⁢i⁢d)

**FIGURE 5:**
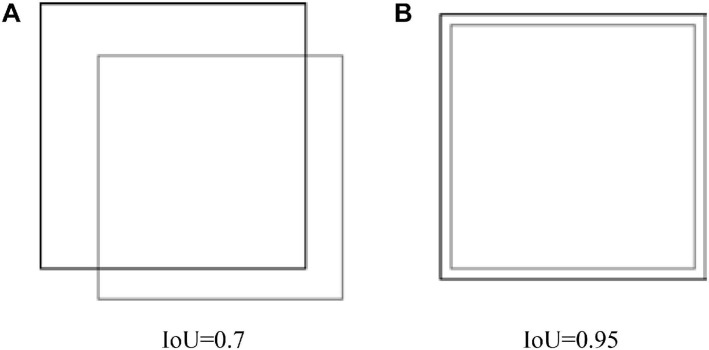
IoU schematic. **(A)** IoU=0.7. **(B)** IoU=0.95.

In the abovementioned formula, the centroid is the bounding box selected as centers in clustering; the box is the bounding box labeled in samples; and IoU(box,centroid) represents the merging ratio of sample annotation boxes and cluster center boxes (intersection over union, IoU), that is, the merging of the intersection ratio of the detection result and the ground truth. As shown in Formula (4) and Figure 5.

(4) $$I\,o\,U=\frac{D\,e\,t\,e\,c\,t\,i\,o\,n\,R\,e\,s\,u\,l\,t\cap G\,r\,o\,u\,n\,d\,T\,r\,u\,t\,h}{D\,e\,t\,e\,c\,t\,i\,o\,n\,R\,e\,s\,u\,l\,t\cup G\,r\,o\,u\,n\,d\,T\,r\,u\,t\,h}$$

(a) IoU = 0.7 (b) IoU = 0.95

When the IOU value is maximum, the annotation box and anchor box match best, d(box,centroid) is minimum, and the annotation box is assigned to the cluster center. Compared with the K-means clustering algorithm, it uses the strategy that the initial center points are as far away from each other as possible as to measure the average overlap degree of the object cluster, so that the clustering results are not affected by the random selection of the initial cluster center point distance, and the clustered prior box is closer to the object box of the dataset.

**FIGURE 6:**
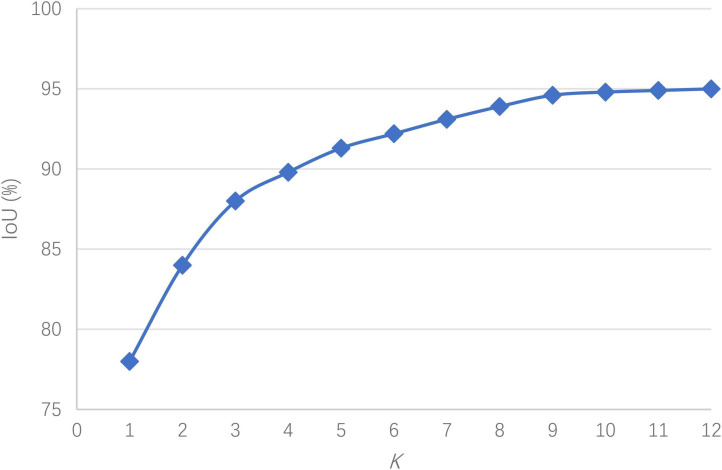
The relationship between the IoU and K.

Let K = 1, 2,…12. Cluster analysis was performed on the dataset samples, and the relationship between the IoU and K was obtained as shown in Figure 6.

It is observed in Figure 6 that when the number of anchor boxes is 9, the average IoU reaches 94.6%, and there is no important improvement thereafter. To balance the IoU and network complexity, the clustering result of K = 9 is taken as the anchor box size in the network, i.e., (52, 20), (65, 29), (73, 32), (84, 36), (89, 40), (97, 46), (109, 58), (122, 63), and (136, 71).

### Multiscale Training

Compared with the YOLO model, the model proposed in this study does not contain a fully connected layer, so it is possible to try different sizes of input images for multiscale training when training convolutional neural networks. By training input images at different scales, the model can achieve the task of adapting to object detection at multiple scales.

**FIGURE 7:**
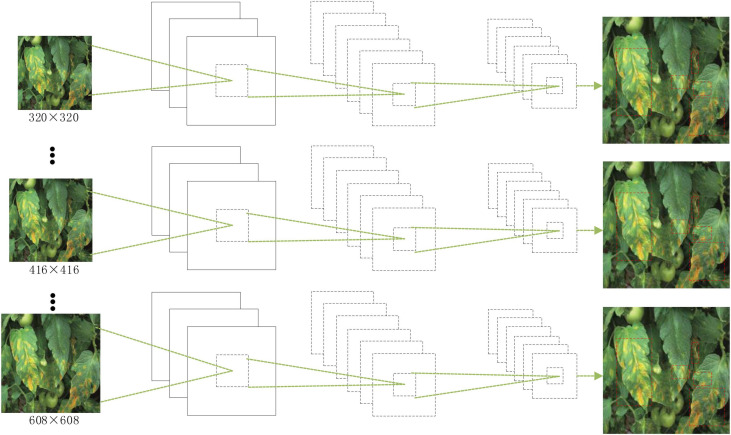
Schematic diagram of the multiscale training process.

Since the improved network contains four residual modules and one dense connection block, the minimum size image is 1/32 of the input image, so when changing the image size, it is necessary to ensure that it is a multiple of 32. Therefore, the image size of the training set is divided into a variety of image scales, which are {320, 352,…, 608}. During training, a scale training is selected randomly 10 times per iteration. The schematic diagram of the multiscale training process is shown in Figure 7.

The multiscale training benefits the robustness of the model by being able to accept any size of image as input and therefore improves the performance indicators of the model network.

## Experiment Design

### Experimental Platform

The experimental environment configuration is as follows: Intel i7-9750H processor, Nvidia GeForce RTX 2060 graphics card, 16-GB memory, and operating system Ubuntu 16.04. The experiment adopts Python programming language, and the deep learning framework is Keras TensorFlow.

### Dataset Construction

Due to the lack of datasets of tomato anomalies in the complex natural environment and because the quality of images is not high, within the context of this study, a tomato anomaly dataset was created by taking photos from complex natural environments and from the Internet as well. The dataset contains 15,000 images of tomato anomalies in various scenarios. Dataset images include occlusion, shading, and other situations. The dataset was converted into VOC2007 dataset format according to the experimental requirements, and the data were annotated with LableImg annotation software.

### Model Training

In the original data set, 70%, 20%, and 10% images of each category were selected to form the training set, validation set, and test set, respectively. In the YOLO-Dense model proposed in this study, the training process uses the trained weight file of the original YOLOv3 as the initialization parameter. Because different network structures need to be trained in the comparative experiment, and the number of iterations to achieve the optimal detection performance is also different, this study monitors dynamically during training and saves the weight file of the network every 1,000 iterations in order to select the best weight file to prevent overfitting. In the iteration process, after 100 and 150 rounds, the learning rate decay factor is set to 0.1, that is, the current learning rate is 0.1 times the previous learning rate. Some experimental training parameter settings that improve the optimal network detection effect are shown in Table 1. At the same time, a multiscale training strategy is used, i.e., random selection of an input image size every 10 rounds to achieve the effect that the model can adapt to image features of different size.

**TABLE 1:** Selection of key parameters.

Parameter name	Parameter value
Batch size	64
Learning rate	0.0026
Decay	0.0054
Momentum	0.9
Factor	0.1

### Evaluating Indicator

The detection accuracy of each category in the detection of tomato anomalies is very important. False-positive and false-negative detection may cause the risk of further spread of the disease. Therefore, average precision (AP) and mean average precision (mAP) were selected as the evaluation indexes of object detection algorithm in this study. These two evaluation indicators take into account the accuracy rate (Precision, P) and recall rate (Recall, R):

(5) $$P\,\left(c\,l\,a\,s\,s\,e\,s\right)=\frac{T\,P}{T\,P+F\,P}$$

(6) $$R\,\left(c\,l\,a\,s\,s\,e\,s\right)=\frac{T\,P}{T\,P+F\,N}$$

(7) $$A\,P=\int_{0}^{1}P\,\left(R\right)\,dR$$

(8) $$m\,A\,P=\frac{\sum_{i=1}^{N}A\,P\,i}{N}$$

Taking the gray mold category in the detection object of this research as an example, TP in the above formula indicates that the detection model detects the correct gray mold object as the number of gray mold diseases, FP indicates the number of false detection of other categories of object as gray mold diseases, and FN indicates the number of false detection of the correct gray mold object as objects of other categories. The values of recall rate and accuracy rate are taken as abscissa and ordinate, respectively, and a P–R curve is drawn, and the area under the curve is AP. For all categories (the number of categories is denoted as N), the average precision is obtained by calculating AP and taking the mean value. mAP is an important index for evaluating the performance of the model, which can reflect the overall performance of the network model and avoid the problem of extreme performance of some categories and weakening the performance of other categories in the evaluation process.

## Experimental Results and Analysis

### Comparison of Algorithm Performance Under Different Resolution Images

**FIGURE 8:**
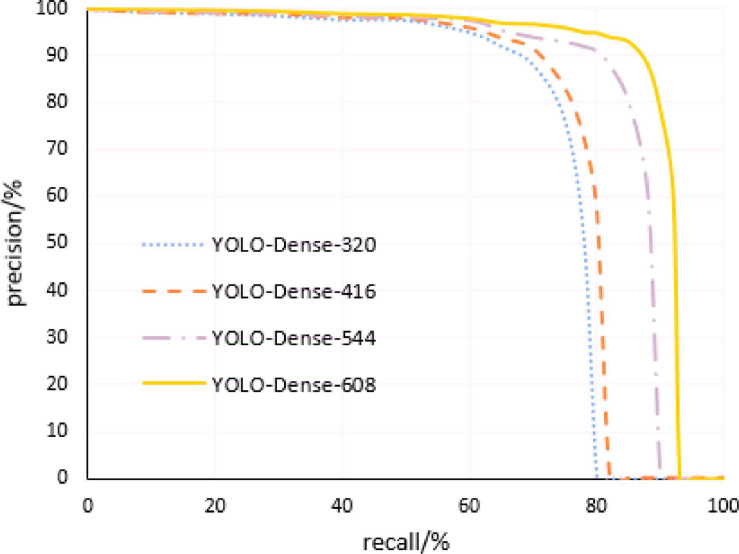
Precision–Recall curves of the proposed model at four different image resolutions.

The multiscale training method makes the model robust to different resolution images. The corresponding models of this study were trained by changing the resolution of the input image to 320 × 320, 416 × 416, 544 × 544, and 608 × 608, respectively. Then, based on the obtained detection model, the test set was tested separately by adjusting the threshold of the comprehensive score of tomato anomaly detection, and the corresponding Precision–Recall curve of each model was obtained. Figure 8 shows the Precision–Recall curve of the model proposed in this study at four different image resolutions, and Table 2 gives the results table of its specific detection evaluation index.

**TABLE 2:** Algorithm performance under different resolutions.

Resolution	mAP (%)	Detection time/(ms)
320 × 320	90.26	17.68
416 × 416	92.32	18.99
544 × 544	96.40	20.27
608 × 608	96.98	29.98

From the above table, we can see that the algorithm performance under different resolution images is satisfying, the mAP value of the model can reach more than 90%, and the detection time of a single image can be controlled within 30 ms. As the resolution of the input image increases, the size of the output feature map, the number of output grids, and the mAP value of the model increase as well; however, the detection speed decreases. When the input resolution is 608 × 608, the detection time of a single image of the model increases to 29.98 ms, affecting the real-time performance of the system. Therefore, it is necessary to select the appropriate resolution for tomato anomaly detection after considering the detection accuracy and detection speed.

### Comparison of Detection Accuracy

The test set is used for testing, and the experimental results are compared with the results of other commonly used object detection algorithms such as the original YOLOv3, Faster R-CNN, and SSD. The results are shown in Table 3.

**TABLE 3:** Comparison of detection accuracy (AP) (%).

Disease/pest	SSD	Faster R-CNN	The original YOLOv3	YOLO-Dense
Early blight	85.46	91.35	90.84	96.71
Late blight	85.67	91.22	90.67	96.68
Yellow leaf curl virus	84.78	91.17	89.98	96.28
Brown spot	85.19	91.08	89.69	93.98
Coal pollution	85.03	91.26	90.52	96.86
Gray mold	84.99	91.41	90.17	96.78
Leaf mold	85.11	90.96	89.65	96.56
Navel rot	85.79	90.89	89.37	96.55
Leaf curl disease	84.98	90.71	89.44	96.48
Mosaic	84.43	90.29	89.32	96.51
Leaf miner	79.12	86.31	85.38	94.95
Greenhouse whitefly	78.01	80.58	79.69	94.01

Compared with Faster R-CNN, SSD, and other algorithms, the YOLO-Dense algorithm has higher detection accuracy in terms of accuracy (AP). Compared with the original YOLOv3, the proposed algorithm in this study achieves an improvement in the average detection accuracy of 12 categories of detection objects. The main reason is that the original YOLOv3 network directly divides an image into 7 × 7 grid, and each grid is predicted with 2 bounding boxes, and each grid predicts only one object, which easily leads to inaccurate positioning. In contrast, as for the YOLO-Dense algorithm, it was mentioned before that during training various image sizes could be used in order for the model to be robust and accurate. Moreover, because the original YOLOv3 network only detects one class for each prediction box, and there are different objects of anomalies in this dataset, the detection result on the closely related Leaf miner and the greenhouse whitefly is not ideal.

Faster R-CNN has high accuracy in large-object recognition, but the accuracy of small-object recognition is very low. The reason is that Faster R-CNN introduces the anchor idea to predict 9 anchor boxes with equal ratio of length to width and area ratio in each position of the feature map, which greatly improves the position prediction ability of the model. However, because Faster R-CNN does not cluster the anchor box, the anchor box for the PASCAL VOC dataset is not applicable to the characteristics of this dataset, resulting in low accuracy of small object detection. On the contrary, the large object is closer to the characteristics of the PASCAL VOC dataset, and the large size diseases are clearly distinguishable, so the detection accuracy can reach more than 90%.

In the actual object detection process of tomato anomalies, the original YOLOv3 algorithm did not perform satisfactorily due to the complex scenes and small differences among different disease classes, especially in the detection of occluded objects and small objects. This study attempts to improve on the basis of the YOLOv3 algorithm and proposes a detection algorithm for tomato anomaly detection task in greenhouse scenarios. Applying DenseNet to the detection of tomato anomalies can improve the expression ability of the network model and thus improve the detection accuracy.

### Comprehensive Performance Comparison

In the case of the low recognition rate of the original YOLO algorithm for tomato anomaly detection, the proposed newly improved network structure YOLO-Dense obtained its optimal performance at 8000 iterations. A comprehensive performance comparison is shown in Table 4.

**TABLE 4:** Comprehensive performance comparison.

Algorithm name	mAP (%)	Time/(ms)	False detection rate (%)	Missed detection rate (%)
SSD	84.32	25.69	1.38	1.29
Faster R-CNN	90.67	2868.94	1.87	1.97
YOLO v3	88.31	21.18	1.05	1.14
YOLO-Dense	**96.41**	**20.28**	**0.61**	**0.96**

*Bold values highlight the performance of the proposed algorithm.*

For the YOLO-Dense network proposed in this study, although the network appears redundant when densely connected, it cannot increase parameters and computation too much, thus not having too much impact on the detection speed. Especially, the concatenation method of dense network connection is adopted, which enables each layer to obtain gradient and input signal directly from loss function, so as to train a deeper network, further improving the detection accuracy of the network, reduce the detection speed, and improve the overall performance of the network. This study also draws on the idea of anchor, which predicts 3 anchor boxes (i.e., 3 × 3 anchor boxes per grid) for multiple scales of YOLO-Dense, so that it is equal to the number of anchor boxes of Faster R-CNN and sets different scales for different sizes of objects to detect, which greatly improves the detection accuracy. The output layer uses the Logistic classifier instead of the original Softmax classifier, which improves the final detection accuracy. It is also seen from Table 4 that the network proposed in this study has the highest average detection accuracy (mAP), the lowest false detection rate, and a missed detection rate, but the detection time is 141 times faster than that of Faster R-CNN. Compared with other algorithms, the speed of Faster R-CNN is the slowest. The biggest difference of Faster R-CNN is that regression and classification are separated. Thus, the detection speed of Faster R-CNN is far behind the other three network frameworks.

By using dense connections, feature fusion and reuse are achieved. By improving the K-means algorithm, object bounding box dimensions are clustered and anchor boxes are calculated for self-made tomato anomaly image datasets; multiscale training is used to enhance the robustness of the model against different sizes. The experimental results show that the YOLO-Dense algorithm improves the detection rate of small objects and occluded objects. Compared with the commonly used algorithms such as SSD, Faster R-CNN, and original YOLOv3, the detection effect is better and the robustness is stronger.

## Conclusion

This study proposes a tomato anomaly detection method based on the deep-learning YOLO framework. Integrating the dense connection idea of DenseNet into the feature extraction part of the original YOLO network realizes the high fusion and multiplexing of feature information. Meanwhile, the improved K-means clustering algorithm is used for anchor box calculation to improve the matching degree between prior anchors and the feature map, so as to adapt to the detection task of tomato anomalies and improve the detection accuracy. The experiment shows that the optimized model has high detection accuracy and fast speed. The model has strong robustness to the detection of tomato anomalies in the complex natural environment, with an average accuracy of 96.41%. Also, the detection time of a single image is only 20.28 ms. The experiment verifies that this method can be used for the detection of tomato anomalies in the complex natural environment. Among its potential applications are in handheld devices, edge computing terminals, and other systems.

In conclusion, compared with the other three algorithms, the YOLO-Dense algorithm has certain advantages in performance. The model makes full use of low-level feature information to improve the detection rate of small objects; dense connection reduces the interference of useless features to the model, realizes feature enhancement, improves the detection effect of occluded objects, and improves the model performance. The experimental results prove the effectiveness of the algorithm.

## Data Availability Statement

The raw data supporting the conclusions of this article will be made available by the authors, without undue reservation.

## Author Contributions

JL and XW conducted the experiments and data analysis, and wrote the manuscript. XW revised the manuscript. Both authors read and approved the manuscript.

## Conflict of Interest

The authors declare that the research was conducted in the absence of any commercial or financial relationships that could be construed as a potential conflict of interest.

## References

[B1] Alemu K. (2015). Detection of diseases, identification and diversity of viruses: a review. *J. Biol. Agric. Healthc.* 5 204–213.

[B2] Arthur D. Vassilvitskii S. (2007). “K-Means++: the advantages of careful seeding,” in *Proceedings of the Eighteenth Annual ACM-SIAM Symposium on Discrete Algorithms, SODA 2007*, (New Orleans, LA: ACM).

[B3] Barbedo J. G. A. (2016). A review on the main challenges in automatic plant disease identification based on visible range images. *Biosyst. Eng.* 144 52–60. 10.1016/j.biosystemseng.2016.01.017

[B4] Chen W. Huang H. Peng S. Zhou C. Zhang C. (2020). Yolo-face: a real-time face detector. *Vis. Comput.* 9. 10.1007/s00371-020-01831-7 [Epub ahead of print].

[B5] Fu L. Feng Y. Wu J. Liu Z. Gao F. Majeed Y. (2020). Fast and accurate detection of kiwifruit in orchard using improved YOLOv3-tiny model. *Precis. Agric.* 1–23. 10.1007/s11119-020-09754-y

[B6] Fuentes A. F. Sook Y. Jaesu L. Sun P. D. (2018). High-performance deep neural network-based tomato plant diseases and pests diagnosis system with refinement filter bank. *Front. Plant Sci.* 9:1162. 10.3389/fpls.2018.01162 30210509 PMC6124392

[B7] Ghazi M. M. Yanikoglu B. Aptoula E. (2017). Plant identification using deep neural networks via optimization of transfer learning parameters. *Neurocomputing* 235 228–235. 10.1016/j.neucom.2017.01.018

[B8] Girshick R. Donahue J. Darrell T. Malik J. (2014). “Rich feature hierarchies for accurate object detection and semantic segmentation,” in *Proceedings of the 2014 IEEE Conference on Computer Vision and Pattern Recognition CVPR*, (Columbus, OH: IEEE).

[B9] He K. Zhang X. Ren S. Sun J. (2014). Spatial pyramid pooling in deep convolutional networks for visual recognition. *IEEE Trans. Pattern Anal. Mach. Intell.* 37 1904–1916. 10.1109/tpami.2015.2389824 26353135

[B10] Heuvelink E. (2005). Greenhouse tomato production. *Crop Prod. Sci. Hortic.* 12 394–399.

[B11] Huang G. Liu Z. Laurens V. D. M. Weinberger K. Q. (2016). “Densely connected convolutional networks,” in *Proceedings of the IEEE Conference on Computer Vision and Pattern Recognition (CVPR)*, (Honolulu, HI: IEEE).

[B12] Juncheng M. Keming D. Feixiang Z. Lingxian Z. Zhihong G. Zhongfu S. (2018). A recognition method for cucumber diseases using leaf symptom images based on deep convolutional neural network. *Comput. Electron. Agric.* 154 18–24. 10.1016/j.compag.2018.08.048

[B13] Li W. Chen P. Wang B. Xie C. (2019). Automatic localization and count of agricultural crop pests based on an improved deep learning pipeline. *Sci. Rep.* 9 1–11. 31065055 10.1038/s41598-019-43171-0 PMC6504937

[B14] Li Y. Han Z. Xu H. Liu L. Zhang K. (2019). Yolov3-lite: a lightweight crack detection network for aircraft structure based on depthwise separable convolutions. *Appl. Sci.* 9:3781. 10.3390/app9183781

[B15] Lin T.-Y. Dollar P. Girshick R. He K. Hariharan B. Belongie S. (2017). *Proceedings of the IEEE Conference on Computer Vision and Pattern Recognition (CVPR).* San Juan, PR: IEEE, 2117–2125.

[B16] Liu J. (2018). Tomato yield estimation based on object detection. *J. Adv. Comput. Intell. Intell. Inform.* 22 1120–1125. 10.20965/jaciii.2018.p1120

[B17] Liu W. Anguelov D. Erhan D. Szegedy C. Berg A. C. (2016). “SSD: single shot multibox detector,” in *Proceedings of the European Conference on Computer Vision*, (Cham: Springer International Publishing).

[B18] Martinelli F. Scalenghe R. Davino S. Panno S. Scuderi G. Ruisi P. (2015). Advanced methods of plant disease detection. a review. *Agron. Sustain. Dev.* 35 1–25.

[B19] Pethybridge S. J. Nelson S. C. (2015). Leaf doctor: a new portable application for quantifying plant disease severity. *Plant Dis.* 99 150420070924008. 10.1094/PDIS-03-15-0319-RE 30690990

[B20] Prasad S. Peddoju S. K. Ghosh D. (2014). “Energy efficient mobile vision system for plant leaf disease identification,” in *Proceedings of the Wireless Communications & Networking Conference*, (Istanbul: IEEE).

[B21] Redmon J. Divvala S. Girshick R. Farhadi A. (2016). “You only look once: unified, real-time object detection,” in *Proceedings of the 2016 IEEE Conference on Computer Vision and Pattern Recognition (CVPR)*, (Las Vegas, NV), 779–788. 10.1109/CVPR.2016.91

[B22] Redmon J. Farhadi A. (2017). “YOLO9000: better, faster, stronger,” in *Proceedings of the IEEE Conference on Computer Vision & Pattern Recognition*, Honolulu, HI, 6517–6525.

[B23] Ren S. He K. Girshick R. Sun J. (2017). Faster r-cnn: towards real-time object detection with region proposal networks. *IEEE Trans. Pattern Anal. Mach. Intell.* 39 1137–1149. 10.1109/tpami.2016.2577031 27295650

[B24] Ren X. Liu X. (2020). Mask wearing detection based on yolov3. *J. Phys. Conf. Ser.* 1678:012089. 10.1088/1742-6596/1678/1/012089

[B25] Shahid N. Kalofolias V. Bresson X. Bronstein M. Vandergheynst P. (2015). “Robust principal component analysis on graphs,” in *Proceedings of the 2015 IEEE International Conference on Computer Vision (ICCV)*, (Santiago: IEEE), 2812–2820.

[B26] Tang Y. Chen M. Wang C. Luo L. Li J. Zou X. (2020). Recognition and localization methods for vision-based fruit picking robots: a review. *Front. Plant Sci.* 11:510. 10.3389/fpls.2020.00510 32508853 PMC7250149

[B27] Xie J. Yu J. Chen B. Feng Z. Siddique K. H. M. (2017). Facility cultivation systems: a chinese model for the planet. *Adv. Agron.* 145 1–42. 10.1016/bs.agron.2017.05.005

[B28] Xing S. Lee M. Lee K. K. (2019). Citrus pests and diseases recognition model using weakly dense connected convolution network. *Sensors* 19:3195. 10.3390/s19143195 31331122 PMC6679302

[B29] Xu G. Zhang F. Shah S. G. Ye Y. Mao H. (2011). Use of leaf color images to identify nitrogen and potassium deficient tomatoes. *Pattern Recognit. Lett.* 32 1584–1590. 10.1016/j.patrec.2011.04.020

[B30] Xu Z. Shou W. Huang K. Zhou S. Li G. Tang G. (2000). The current situation and trend of tomato cultivation in china. *Acta Physiol. Plant.* 22 379–382. 10.1007/s11738-000-0061-y

[B31] Xu Z. F. Jia R. S. Sun H. M. Liu Q. M. Cui Z. (2020). Light-yolov3: fast method for detecting green mangoes in complex scenes using picking robots. *Appl. Intell.* 50 1–18.

[B32] Zhang B. Wang G. Wang H. Xu C. Li Y. Xu L. (2021). Detecting small chinese traffic signs via improved yolov3 method. *Math. Probl. Eng.* 2021 1–10. 10.1155/2021/8826593

[B33] Zhang X. Gao Y. Wang H. Wang Q. (2020). Improve yolov3 using dilated spatial pyramid module for multi-scale object detection. *Int. J. Adv. Robot. Syst.* 17:172988142093606. 10.1177/1729881420936062

[B34] Zhang Y. Song C. Zhang D. (2020). Deep learning-based object detection improvement for tomato disease. *IEEE Access* 8:56607–56614. 10.1109/access.2020.2982456

